# Association of Thrombomodulin Expression with Bladder Cancer Grade, Stage, and Recurrence: A Single-Center Prospective Pilot Study 

**DOI:** 10.30699/ijp.2025.2047132.3387

**Published:** 2025-07-01

**Authors:** Mohammad Reza Nikoobakht, Shima Esmaeilpanah, Iman Menbari Oskouie, Mahdi Khoshchehreh, Farshid Alaeidini, Seyed Mohammad Kazem Aghamir

**Affiliations:** 1 Urology Research Center, Tehran University of Medical Sciences, Tehran, Iran; 2 Department of Pathology, University of California, Los Angeles, USA; 3 Tehran Heart Center, Cardiovascular Diseases Research Institute, Tehran University of Medical Sciences, Tehran, Iran

**Keywords:** Thrombomodulin; Bladder cancer; Recurrence; Immunohistochemistry

## Abstract

**Background & Objective:**

Bladder cancer is the fourth most prevalent malignancy and lacks reliable biomarkers for predicting tumor stage, grade, and clinical outcomes. This study aimed to evaluate the association between thrombomodulin (TM)–positive cell rate (PR) and tumor grade, stage, and recurrence in patients with bladder cancer.

**Methods:**

This prospective observational pilot study was conducted at the Urology Clinic of Sina Hospital, Tehran, between March and December 2022. A total of 51 patients diagnosed with bladder cancer following cystoscopy and transurethral resection of bladder tumor (TURBT) were enrolled. Of these, 11 patients with stage T2 disease underwent radical cystectomy. TM expression was assessed by immunohistochemical staining, and the PR score was calculated. The remaining 40 patients underwent follow-up cystoscopy 3 months post-TURBT to assess for recurrence or progression. Statistical analyses were performed using SPSS version 26, with comparisons of quantitative variables conducted using ANOVA and t tests.

**Results:**

The mean age of participants was 66.73 ± 11.00 years, and 46 were male. The mean TM PR value was 25.51 ± 6.24. No significant differences in TM PR values were observed among different tumor grades (p = 0.144) or stages (p = 0.815). Additionally, there were no significant differences in TM PR values or PR scores between patients with and without recurrence at 3-month follow-up cystoscopy (p = 0.144 and p = 0.085, respectively).

**Conclusion:**

TM PR values did not correlate with tumor grade, stage, or recurrence in this cohort of bladder cancer patients. Further studies with larger sample sizes and longer follow-up periods are warranted.

## Introduction

Bladder cancer is diagnosed in over 430,000 individuals worldwide each year, ranking as the fourth most common cancer in men and the eleventh in women (1,2). Diagnosis is typically confirmed through histopathological evaluation of tissue obtained via transurethral resection of the bladder tumor (TURBT) (3). While most urothelial carcinomas (UCs) are detected at an early stage, approximately 25% present at an advanced stage (2). In aggressive UC variants with poor clinical outcomes, there is an urgent need for sensitive and specific immunohistochemical (IHC) markers—or combinations of markers—to confirm urothelial origin and guide treatment decisions (4,5).

Prostate-specific antigen (PSA) has long served as a pioneering biomarker in prostate cancer diagnosis and prognosis, revolutionizing urology and oncology since its discovery in the 1970s. PSA testing has significantly improved early detection, disease monitoring, and risk stratification in prostate cancer patients (6). Beyond PSA, other biomarkers such as CXCR2 and CXCR3 have demonstrated diagnostic and prognostic utility in urinary tract malignancies, including renal cell carcinoma (7).

IHC markers are essential for distinguishing urothelial carcinoma from other malignancies and for identifying the primary site of metastatic tumors. Commonly used markers include CK7, CK20, p63, thrombomodulin, GATA3, and Uroplakin II, all of which are helpful in confirming the urothelial origin of tumors (8,9). Additional markers like 34βE12 (a high-molecular-weight cytokeratin) assist in differentiating benign from malignant lesions through basal cell identification, while alpha-methylacyl-CoA racemase (AMACR) aids in detecting malignant prostate or urothelial lesions due to its overexpression in neoplastic glands (10–12).

Thrombomodulin (TM) is a transmembrane protein primarily expressed by vascular endothelial cells, playing a central role in coagulation by enhancing protein C activation (13–15). Although TM is a sensitive marker for urothelial cells, its specificity is limited (16), as it is also expressed in various other cell types, including mesothelial cells (17,18), keratinocytes (19,20), syncytiotrophoblasts (21), synovial cells (22), mesangial cells of the kidney (23), and platelets (24).

Currently, bladder cancer lacks clinically validated biomarkers for accurately predicting disease stage and clinical outcomes (25). Therefore, there is a substantial need to identify reliable biomarkers capable of assessing tumor aggressiveness or stratifying recurrence risk in patients with bladder cancer (26). Although a few studies have examined the relationship between TM expression and bladder cancer characteristics, their findings have been inconsistent.

The present study aimed to evaluate the association between TM expression and the grade and stage of bladder cancer. Additionally, we sought to determine whether TM could serve as a useful laboratory marker for predicting recurrence, thereby supporting clinical decision-making. The overarching objective was to assess the potential of thrombomodulin as a prognostic biomarker to aid in predicting outcomes and improving patient management in bladder cancer.

## Materials and Methods

### Patients

This observational, prospective pilot study was conducted following approval from the Institutional Review Board (IRB) at Tehran University of Medical Sciences (IR.TUMS.SINAHOSPITAL.REC.1401.033). The study was carried out at the Urology Clinic of Sina Hospital, a tertiary care center located in Tehran, Iran. A total of fifty-one patients, who were clinically diagnosed with bladder cancer, were enrolled in the study between March and December 2022. All cases included in the study were histologically confirmed as transitional cell carcinoma (TCC). The histopathological classification was confirmed through cystoscopy and biopsy, with all specimens being evaluated by experienced pathologists at the hospital’s pathology department. 

Patients provided informed written consent for the collection and storage of their medical information and tissue samples. Clinical data, including patient demographics, treatments, and outcomes, were obtained from medical records. Subsequently, Cystoscopy and TURBT procedures were performed. Radical cystectomy was conducted for eleven patients with T2 pathological stage. Three months after TURBT, the remaining 40 patients underwent cystoscopy to assess for recurrence and progression.

The primary tumor's pathological stage was determined based on the 2010 American Joint Committee on Cancer TNM criteria: Stage I (T_1_N_0_M_0_), Stage II (T_2_N_0_M_0_), Stage III (T_3_N_0_M_0_ and T_4a_N_0_M_0_), and Stage IV (T_4b_N_0_M_0_, T_any_N_1-3_M_0_, and T_any_N_any_M_1_) (27). 

**Figure 1:**
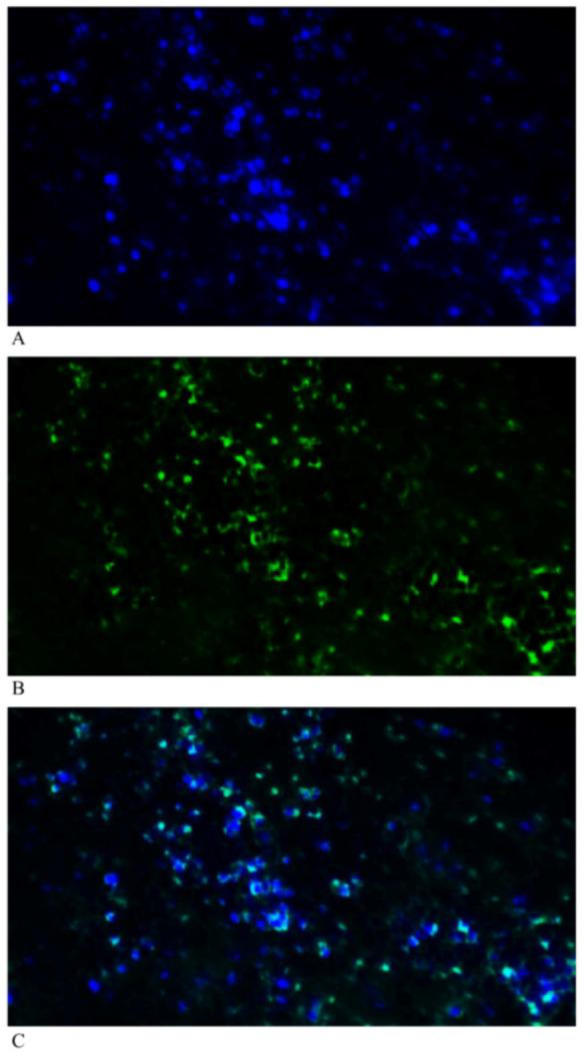
Immunohistochemistry staining, (A) DAPI (B) Thrombomodulin (C) Merged of DAPI and thrombomodulin

### Immunohistochemistry

Immunohistochemical (IHC) staining was performed on tissue microarray (TMA) sections. The TMA was constructed using 1-mm tissue cores, with two cores per patient, prepared using a manual tissue arrayer (Beecher Instruments, Silver Spring, MD). Thrombomodulin expression was assessed using a monoclonal antibody (clone 1009, dilution 1:10; Dako).

Briefly, 4-μm-thick paraffin-embedded sections were deparaffinized in xylene and rehydrated through a graded series of ethanol. Staining was conducted using a BOND-MAX autostainer (Leica Biosystems). Slides were incubated with the primary thrombomodulin antibody, followed by application of a visualization reagent containing secondary goat anti-mouse immunoglobulin and horseradish peroxidase (HRP) conjugated to a dextran polymer backbone. Chromogenic detection was performed using 3,3′-diaminobenzidine (DAB), and slides were counterstained with Mayer’s hematoxylin.

Representative thrombomodulin-stained slides are shown in Figure 1.

## Results

A total of 51 patients with bladder cancer were included in the study, with a mean age of 66.73 ± 11.00 years. Of these, 46 were male. Among the participants, 29 had low-grade tumors, and 20 patients presented with recurrent bladder cancer. The mean thrombomodulin (TM) positive rate (PR) value was 25.51 ± 6.24. In terms of TM PR scoring, 11 patients had a score of 1, while the remaining patients had a score of 2 (Table 1).

Table 2 presents the relationship between tumor characteristics and TM PR values. No significant differences in TM PR values were observed among patients with different tumor grades (p = 0.144) or tumor stages (p = 0.815). Additionally, there were no significant differences in either TM PR values or TM PR scores between patients with and without recurrence on cystoscopy at 3 months post-TURBT (p = 0.144 and p = 0.085, respectively).

Moreover, a multivariable logistic regression analysis was performed to evaluate the relationship between TM PR values and three-month post-TURBT cystoscopy results as well as tumor grade, while adjusting for age, BMI, and admission status (new case vs. recurrence). The analysis revealed no significant associations (Table 3).

**Table 1:** Clinicopathological Characteristics

Variable		Mean ± SD / n (%)
Age	Years	66.73 ± 11.00
BMI	Kg/m^2^	24.31 ± 3.42
Gender	Male	46 (90.2%)
	Female	5 (9.8%)
Tumor grade	Low grade	29 (56.9%)
	High grade	22 (43.1%)
Tumor Stage	Ta	12 (23.5%)
	T1	28 (54.9%)
	T2	11 (21.6%)
Admission status	New case	31 (60.8%)
	recurrence	20 (39.2%)
Cystoscopy (three months later)	Normal	30 (75.0%)
	recurrence	10 (25.0%)
TM PR score	0	0 (0%)
	1	11 (21.6%)
	2	40 (78.4%)
	3	0 (0%)
TM PR value		25.51 ± 6.24

TM: thrombomodulin, PR: positive cell rate

**Table 2:** Relation of tumor characteristics and TM PR

Variable		TM PR value (Mean ± SD)	p-value
Tumor grade	Low grade	26.62 ± 6.81	0.144
	High grade	24.04 ± 5.18	
Tumor Clinical Stage	Ta	26.53 ± 6.48	0.815
	T1	25.19 ± 6.94	
	T2	25.19 ± 3.61	
Cystoscopy (three months later)	Normal	26.41 ± 6.74	0.144
	recurrence	22.75 ± 6.61	

TM: thrombomodulin, PR: positive cell rate

**Table 3:** Relation between tumor characteristics and TM PR using logistic regression

Variable	Odds Ratio	95% CI	p-value
Tumor grade	0.932	0.847 – 1.025	0.146
Cystoscopy (three months later)	0.916	0.813 – 1.032	0.148

## Discussion

This study offers a distinct perspective by focusing on Iranian bladder cancer patients treated with TURBT and cystectomy, in contrast to previous studies conducted in other populations (29,30). In Iran, the standard approach to bladder cancer predominantly involves surgical treatments such as TURBT for non–muscle-invasive cases and radical cystectomy for muscle-invasive bladder cancer (MIBC), with limited use of bladder-sparing therapies (31). This treatment consistency may facilitate more reliable associations between tumor stage, grade, and recurrence outcomes. Moreover, the Iranian population—underrepresented in global research—presents unique genetic, environmental, and healthcare-related factors that could influence tumor biology and clinical outcomes. As such, evaluating the prognostic utility of biomarkers like thrombomodulin (TM) within this context is warranted.

In this observational pilot study, TM PR values did not significantly differ across tumor grades (p = 0.144) or stages (p = 0.815). Similarly, no significant difference was found in TM PR values or PR scores between patients with and without recurrence, as assessed by cystoscopy three months post-TURBT (p = 0.144 and p = 0.085, respectively).

In a prospective single-center pilot study by Tongwiis et al. involving 35 patients, TM expression was significantly reduced in bladder tumor tissues compared with normal bladder tissues (p = 0.010). Consistent with our findings, no significant association was observed between TM expression and tumor grade or two-year recurrence (p = 0.83 and p = 0.89, respectively). However, unlike our results, they identified a significant correlation between TM expression and cancer stage (p = 0.039) (29).

Watt et al. investigated TM immunoexpression in 98 individuals using IHC, analyzing tissue cores from transitional cell carcinoma (TCC), squamous cell carcinoma (SCC), adenocarcinoma, sarcoma, and normal tissues. TM scores were higher in TCC and SCC compared with adenocarcinoma and sarcoma (30). Moreover, TM expression decreased across increasing tumor stage and grade, and lower survival rates were observed in patients with TM scores below 3.0. These findings support previous research suggesting that TM may serve as a positive prognostic marker, independent of tumor grade or stage (16,32). Conversely, Parker et al. found no significant difference in TM expression in noninvasive tumors and reported a sensitivity of 69% and specificity of 96% for urothelial lesions using anti-TM antibodies (33). Similarly, Mhawech et al. reported TM expression in 87% of urothelial carcinoma cases—comparable to the 100% expression observed in our cohort—and noted a sensitivity of 48.9% and specificity of 100% (34).

In another study, plasma and urinary TM levels were evaluated in 57 bladder cancer patients and 10 healthy controls using ELISA. Urinary TM levels were significantly reduced, while plasma TM levels were elevated in patients with bladder cancer. Notably, SCC patients had higher plasma TM levels compared with those with TCC. The sensitivity and specificity were 90% and 86% for urinary TM and 76% and 80% for plasma TM, respectively (35).

Previous studies have also proposed a protective role for TM in cancer. Reduced TM expression has been linked to poor prognosis in metastatic lung, breast, and colorectal cancers (36,37). Although the molecular mechanisms underlying this effect are not fully understood, one hypothesis posits that TM downregulation promotes tumor cell migration by increasing vimentin expression and decreasing E-cadherin expression (38,39).

The relatively small sample size is a primary limitation of this study. Larger, multicenter studies are necessary to validate our findings and establish a more robust and generalizable biomarker profile for bladder cancer. Future research should incorporate simultaneous evaluation of TM tissue expression, serum levels, and urinary biomarkers. This comprehensive approach could provide more definitive evidence to support TM’s role in diagnosis and prognosis. Additionally, incorporating patient demographic factors such as family history and educational background may offer further insight into the influence of sociocultural variables on immune status and tumor marker expression.

## Conclusion

This study indicates that there was no significant correlation between the TM PR values and the grade, stage, or recurrence of bladder cancer. However, this observation warrants further investigation with larger sample sizes and extended follow-up periods.

## References

[1] Bray F, Ferlay J, Soerjomataram I, Siegel RL, Torre LA, Jemal A (2018). Global cancer statistics 2018: GLOBOCAN estimates of incidence and mortality worldwide for 36 cancers in 185 countries. CA: a cancer journal for clinicians.

[2] Kamat AM, Hahn NM, Efstathiou JA, Lerner SP, Malmström P-U, Choi W (2016). Bladder cancer. Lancet.

[3] Richterstetter M, Wullich B, Amann K, Haeberle L, Engehausen DG, Goebell PJ (2012). The value of extended transurethral resection of bladder tumour (TURBT) in the treatment of bladder cancer. BJU international.

[4] Willis DL, Porten SP, Kamat AM (2013). Should histologic variants alter definitive treatment of bladder cancer?. Curr Opin Urol.

[5] Lopez Beltran A, Montironi R, Cheng L (2014). Microcystic urothelial carcinoma: morphology, immunohistochemistry and clinical behaviour. Histopathology.

[6] Ilic D, Djulbegovic M, Jung JH, Hwang EC, Zhou Q, Cleves A (2018). Prostate cancer screening with prostate-specific antigen (PSA) test: a systematic review and meta-analysis. bmj.

[7] Bayat A-A, Sadeghi N, Fazli G, Nowroozi MR, Moghadam SO, Radmanesh A (2022). Diagnostic and Therapeutic Implications of Sortilin Expressed on the Surface of Bladder Carcinoma Cells. Iran J Pathol.

[8] Moch H, Cubilla AL, Humphrey PA, Reuter VE, Ulbright TM (2016). The 2016 WHO classification of tumours of the urinary system and male genital organs-part A: renal, penile, and testicular tumours. European urology.

[9] Amin MB, Trpkov K, Lopez-Beltran A, Grignon D, Group IIiDUP (2014). Best practices recommendations in the application of immunohistochemistry in the bladder lesions: report from the International Society of Urologic Pathology consensus conference. Am J Surg Pathol.

[10] Walter B, Weiss T, Hofstädter F, Gaumann A, Hartmann A, Rogenhofer S (2013). Utility of immunohistochemistry markers in the interpretation of post-high-intensive focussed ultrasound prostate biopsy cores. World J Urol.

[11] Rezakhaniha B, Pour NA, Siroosbakhat S (2010). Effect of cystoscopy on prostate-specific antigen, new words about old subject. Int J Cancer Manag.

[12] Sirousbakht S, Rezakhaniha B (2018). Effect of colonoscopy on prostate-specific antigen; new words about an old subject. Int J Cancer Manag.

[13] Boron M, Hauzer-Martin T, Keil J, Sun X-L (2022). Circulating thrombomodulin: release mechanisms, measurements, and levels in diseases and medical procedures. TH Open.

[14] Ito T, Thachil J, Asakura H, Levy JH, Iba T (2019). Thrombomodulin in disseminated intravascular coagulation and other critical conditions-a multi-faceted anticoagulant protein with therapeutic potential. Crit Care.

[15] Giri H, Panicker SR, Cai X, Biswas I, Weiler H, Rezaie AR (2021). Thrombomodulin is essential for maintaining quiescence in vascular endothelial cells. Proc Natl Acad Sci U S A.

[16] Wu C-T, Chang Y-H, Lin P-Y, Chen W-C, Chen M-F (2014). Thrombomodulin expression regulates tumorigenesis in bladder cancer. BMC Cancer.

[17] Yang Y, Cheng B-J, Lu S (2017). Thrombomodulin regulates doxorubicin sensitivity through epithelial-mesenchymal transition in non-small cell lung cancer. Eur Rev Med Pharmacol Sci.

[18] Kondo T, Takahashi M, Yamasaki G, Sugimoto M, Kuse A, Morichika M (2021). Immunohistochemical analysis of thrombomodulin expression in myocardial tissue from autopsy cases of ischemic heart disease. Legal Medicine.

[19] Cheng T-L, Lai C-H, Chen P-K, Cho C-F, Hsu Y-Y, Wang K-C (2015). Thrombomodulin promotes diabetic wound healing by regulating toll-like receptor 4 expression. J Invest Dermatol.

[20] Aboalkher MM, Elsayed SK, Sayed SS, Galal SA (2021). Thrombomodulin in skin diseases. J Recent Adv Med.

[21] Han C, Dong J-f (2021). Saving placental thrombomodulin. Blood.

[22] Nozaki Y, Ri J, Sakai K, Niki K, Funauchi M, Matsumura I (2020). Protective effects of recombinant human soluble thrombomodulin on lipopolysaccharide-induced acute kidney injury. International Journal of Molecular Sciences.

[23] van Aanhold CC, Dijkstra KL, Bos M, Wolterbeek R, van den Berg BM, Bruijn JA (2021). Reduced glomerular endothelial thrombomodulin is associated with glomerular macrophage infiltration in diabetic nephropathy. Am J Pathol.

[24] Watanabe-Kusunoki K, Nakazawa D, Ishizu A, Atsumi T (2020). Thrombomodulin as a physiological modulator of intravascular injury. Front Immunol.

[25] Abogunrin F, O'Kane HF, Ruddock MW, Stevenson M, Reid CN, O'Sullivan JM (2012). The impact of biomarkers in multivariate algorithms for bladder cancer diagnosis in patients with hematuria. Cancer.

[26] Ye F, Wang L, Castillo-Martin M, McBride R, Galsky MD, Zhu J (2014). Biomarkers for bladder cancer management: present and future. American journal of clinical and experimental urology.

[27] Sb E, Dr B, CC C, AG F, FL G (2010). AJCC cancer staging manual. New York: Springer.

[28] Martin FA, McLoughlin A, Rochfort KD, Davenport C, Murphy RP, Cummins PM (2014). Regulation of thrombomodulin expression and release in human aortic endothelial cells by cyclic strain. PLoS One.

[29] Ella-Tongwiis P, Lamb RM, Makanga A, Shergill I, Hughes SF (2020). The role of antibody expression and their association with bladder cancer recurrence: A single-centre prospective clinical-pilot study in 35 patients. BMC urology.

[30] Watt J, Maguire DG, Reid CN, Lamont JV, Fitzgerald SP, Ruddock MW Thrombomodulin expression in bladder cancer tissue and its association with prognosis and patient survival. Research and Reports in Urology.

[31] Kalan Farmanfarma K, Mahdavifar N, Salehiniya H Bladder cancer in Iran: an epidemiological review. Research and Reports in Urology.

[32] Chang Y-J, Cheng Y-W, Lin R-K, Huang C-C, Chen WT-L, Ke T-W (2016). Thrombomodulin influences the survival of patients with non-metastatic colorectal cancer through epithelial-to-mesenchymal transition (EMT). PLoS One.

[33] Parker DC, Folpe AL, Bell J, Oliva E, Young RH, Cohen C (2003). Potential utility of uroplakin III, thrombomodulin, high molecular weight cytokeratin, and cytokeratin 20 in noninvasive, invasive, and metastatic urothelial (transitional cell) carcinomas. The American journal of surgical pathology.

[34] Mhawech P, Uchida T, Pelte M-F (2002). Immunohistochemical profile of high-grade urothelial bladder carcinoma and prostate adenocarcinoma. Hum Pathol.

[35] Refaat LA, Ali OE, Hassan AA, Metwally AM (2012). Urinary thrombomodulin is down regulated in schistosomiasis associated bladder cancer. J Genet Eng Biotechnol.

[36] Hanly A, Redmond M, Winter D, Brophy S, Deasy J, Bouchier-Hayes D (2006). Thrombomodulin expression in colorectal carcinoma is protective and correlates with survival. Br J Cancer.

[37] Karimaei S, Mashhadi R, Mirzaei A, Deyhimfar R, Shabestari AN, Rahimnia R (2022). Antibacterial and antibiofilm activities of nisin from Lactococcus lactis and alteration of the bacteria-induced pro-inflammatory responses on kidney and bladder tumor cell lines. Transl Res Urol.

[38] Tai C-J, Cheng C-W, Su H-Y, Chen W-Y, Wu C-T, Lin F-Y (2014). Thrombomodulin mediates the migration of cervical cancer cells through the regulation of epithelial-mesenchymal transition biomarkers. Tumour Biol.

[39] Fallah B, Barikzaei P, Barikzehi M, Khalili N, Nasiriani K, BagherAbadi M (2022). Effect of telenursing on life quality and care burden of caregivers in patients undergoing bladder tumor resection through duct. Transl Res Urol.

